# Higher Ammonium Transamination Capacity Can Alleviate Glutamate Inhibition on Winter Wheat (*Triticum aestivum* L.) Root Growth under High Ammonium Stress

**DOI:** 10.1371/journal.pone.0160997

**Published:** 2016-08-11

**Authors:** Feng Wang, Jingwen Gao, Yang Liu, Zhongwei Tian, Abid Muhammad, Yixuan Zhang, Dong Jiang, Weixing Cao, Tingbo Dai

**Affiliations:** Key Laboratory of Crop Physiology, Ecology and Production Management, Nanjing Agricultural University, Nanjing, Jiangsu Province, 210095, P. R. China; Institute of Genetics and Developmental Biology Chinese Academy of Sciences, CHINA

## Abstract

Most of the studies about NH_4_^+^ stress mechanism simply address the effects of free NH_4_^+^, failing to recognize the changed nitrogen assimilation products. The objective of this study was to elucidate the effects of glutamate on root growth under high ammonium (NH_4_^+^) conditions in winter wheat (*Triticum aestivum* L.). Hydroponic experiments were conducted using two wheat cultivars, AK58 (NH_4_^+^-sensitive) and Xumai25 (NH_4_^+^-tolerant) with either 5 mM NH_4_^+^ nitrogen (AN) as stress treatment or 5 mM nitrate (NO_3_^-^) nitrogen as control. To evaluate the effects of NH_4_^+^-assimilation products on plant growth, 1 μM L-methionine sulfoximine (MSO) (an inhibitor of glutamine synthetase (GS)) and 1 mM glutamates (a primary N assimilation product) were added to the solutions, respectively. The AN significantly reduced plant biomass, total root length, surface area and root volume in both cultivars, but less effect was observed in Xumai25. The inhibition effects were alleviated by the application of MSO but strengthened by the application of glutamate. The AN increased the activities of GS, glutamate dehydrogenase (GDH) in both cultivars, resulting in higher glutamate contents. However, its contents were decreased by the application of MSO. Compared to AK58, Xumai25 showed lower glutamate contents due to its higher activities of glutamic-oxaloacetic transaminase (GOT) and glutamic-pyruvic transaminase (GPT). With the indole-3-acetic acid (IAA) contents decreasing in roots, the ratio of shoot to root in IAA was increased, and further increased by the application of glutamate, and reduced by the application of MSO, but the ratio was lower in Xumai25. Meanwhile, the total soluble sugar contents and its root to shoot ratio also showed similar trends. These results indicate that the NH_4_^+^-tolerant cultivar has a greater transamination ability to prevent glutamate over-accumulation to maintain higher IAA transport ability, and consequently promoted soluble sugar transport to roots, further maintaining root growth.

## Introduction

Ammonium (NH_4_^+^) and nitrate (NO_3_^-^) are the two major nitrogen (N) forms uptaken by higher plants. In recent years, large amount of N fertilizer has been applied in the agricultural ecosystem resulting in a series of problems, such as the decline in farmland quality, lower nitrogen use efficiency (NUE) and some environmental critics [1–3]. Strategies reducing the amount of N fertilizer or improving the fertilization management could make a contribution to the reduction in N losses, increase in NUE and crop yield [4]. In the fields of terrestrial crops, a terrific amount of applied N fertilizers such as urea is rapidly converted into NO_3_^-^ which easily leaches to the groundwater with irrigation or rainfall onset, resulting in an additional soil N losses and reducing the soil N supplying capacity [5]. In contrast, some recent studies have reported that NH_4_^+^-form N fertilizers combined with nitrification inhibitors can effectively reduce the N losses [6]. Thus reducing the N conversion and maintaining a high concentration of NH_4_^+^ in the soil may be the key tactic to improve the NUE. In China, a large amount of N fertilizer is applied as the base fertilizer, which along with the atmospheric NH_4_^+^ deposition and slow-release N fertilizer leads to short term high NH_4_^+^ concentrations in the soil, which can exceed up to 20 mM [7], much higher than the optimum NH_4_^+^ concentrations (0.1 to 0.5 mM) for the terrestrial crops [8,9]. These contexts of NH_4_^+^ in the soil result in high NH_4_^+^ stress for the crops. In future, under abrupt anthropogenic N inputs, NH_4_^+^ stress will be an alarming universal productive limiting factor in a wide range of crops. Therefore, it is necessary to explore the regulatory mechanisms of NH_4_^+^ stress on the plant growth to increase the crop growth and yields.

Excess NH_4_^+^ causes the deleterious effects ranging from altered plant communities to suppressed growth, reduced productivity even the plant death [10]. The most visible phenotypic characters of NH_4_^+^ stress are represented by the inhibited growth of roots, including shortened roots and reduced gravitropism [3] as reported in some previous studies. As the main organ for capturing nutrients and water, the plant root system has strong morphologic plasticity for changing soil environments. Indole-3-acetic acid (IAA) is a signaling hormon that plays an essential role in regulating and modulating the formation of architecture of root systems, and mainly is synthesized in the young shoot organs and transported from shoots to roots through the phloem, regulating the development of the quiescent center, root cap, root apical meristem, and root vascular differentiation [11]. Under high NH_4_^+^ conditions, the endogenous contents of IAA are usually decreased as the primary root of Auxin Resistant 1 (*aux1*), an auxin influx carrier was found to be as sensitive to root-supplied NH_4_^+^. Nevertheless, there are little reports addressing the reasons for the changes in concentrations of IAA as well as the regulatory mechanism of root morphology under NH_4_^+^ stress.

The formation of roots may also be regulated by the carbohydrate produced by photosynthesis from the above ground parts. Since N assimilation occurs rapidly and vastly when NH_4_^+^ is the sole N source, the consumption of carbon for NH_4_^+^-fed plants is much higher compared to NO_3_^-^-fed plants [12,13], resulting in an intense competition between growth and development of root system and N assimilation on the consumption of carbon. Therefore, under NH_4_^+^ condition the plants need a higher carbon supply to maintain plant root growth. It was reported that high IAA in roots could promote carbohydrate transport from shoots to roots, stimulating root growth. Unfortunately, the regulatory mechanism remained unclear.

Several physiological mechanisms about NH_4_^+^ stress have been suggested as follows: rhizosphere acidification and cytosolic pH disturbance caused by the uptake of NH_4_^+^ [10,14,15]; shortage of essential ions, such as K^+^, Ca^2+^ and Mg^2+^ [16]; damage to the photosynthetic system [17]; shifts in several metabolites levels, such as carbohydrates, amino acids or organic acids [18,19]; imbalance of hormones [10,20] and the futile cycling of NH_4_^+^ increasing the energy-costs [21]. However, not a single mechanism mentioned above can provide an adequate explanation for NH_4_^+^ toxicity or tolerance [3]. NH_4_^+^ tolerant or sensitive cultivars may possess variable metabolic adaptations and tolerance mechanisms in response to NH_4_^+^ stress, which are likely to contribute differently to their adaptation capability to NH_4_^+^ stress. Besides, most of the NH_4_^+^ stress studies focus on estimation of the concentration of free NH_4_^+^ in the plant tissues lacking to address the alterations in primary N assimilation mechanism and its products, which may play an important regulatory role in plant growth under NH_4_^+^ condition.

Ammonium assimilation into amino acids occurs primarily through glutamine synthetase/glutamate synthase (GS/GOGAT; GS, EC 6.3.1.2; GOGAT, EC 1.4.1.14) [4,22,23]. Meanwhile, glutamate dehydrogenase (GDH, EC 1.4.1.2) catalyzes the amination of α-ketoglutarate to glutamate [24,25], in addition to the GS/GOGAT pathway in NH_4_^+^ assimilation. It was detected that activities of GS and GDH were higher under high NH_4_^+^ condition, and it might contribute to an increased tolerance to NH_4_^+^ stress [26]. On the other side, the increased NH_4_^+^ assimilation capability resulted in obvious changes to the levels of nitrogenous compounds, such as amino acids, proteins or polyamines [18,22], and one of them, glutamate occupies an important position in N metabolism [27]. Some evidences showed that glutamate could act as an important signal to modulate root growth through monitoring changes in auxin distribution in plant tissues. High glutamate contents inhibited mitotic activity and cell elongation in the root tip associated with the interrupted transport of auxin from shoots to roots. However, most of studies just focus on the roles of exogenous glutamate in root growth, neglecting the functions of endogenous glutamate. Furthermore, the a-amino group of glutamate may be transferred to other amino acids by transamination, and catalyzed by glutamic-oxaloacetic transaminase (GOT, EC 2.6.1.1) and glutamic-pyruvic transaminase (GPT, EC 2.6.1.2) [27], which is a principal pathway of amino acids transamination and an efficient way that accounts for the relative stability of the endogenous glutamate concentrations. There were few reports about the changes of endogenous glutamate and its roles in shaping architecture of root systems associated with the alterations of IAA under NH_4_^+^ stress. Moreover, previous achievements concerning NH_4_^+^ toxicity and detoxification have been mainly documented in some model organisms such as *Arabidopsis*, rice and mutants plant species [3,28]. Very few reports about the NH_4_^+^ toxicity and tolerance capability in wheat cultivars are available.

The objectives of the present study were to investigate the effects of the nitrogen assimilation product, glutamate, on root growth under high ammonium (NH_4_^+^) conditions in winter wheat in different wheat cultivars i.e. Xumai25 (tolerant to NH_4_^+^-stress) and AK58 (sensitive to NH_4_^+^-stress) in terms of roots morphology to NH_4_^+^ conditions and elucidate the regulatory mechanism. The findings of the study would be capable of exploring the involvement of possible physiological mechanisms in NH_4_^+^ tolerance of wheat cultivars, which would be helpful for the research tasks for selecting and breeding the cultivars having higher adaptability to NH_4_^+^-N nutrition.

## Materials and Methods

### Plants growth conditions and experimental design

Two wheat cultivars selected on the basis of their contrasting attitudes towards NH_4_^+^ stress (Xumai25 as tolerant to NH_4_^+^-stress and AK58 as sensitive to NH_4_^+^-stress) were used as plant material for this study. These cultivars possess similar growth patterns under normal growth conditions but behave differently under high NH_4_^+^ conditions as observed in our pre-experiments (We selected AK58 and Xumai25 on the basis of the changes of dry weight, plant height, root length, etc., but data was not shown in this study). Uniform seeds of both cultivars were selected, surface sterilized with 20% (v/v) H_2_O_2_ for 10 min, rinsed with distilled water, and then germinated in culture dishes covered with wet sterilized gauze until the seed bud length was about 1 cm and then cultivated in sterilized silica sand. After germination, when seedlings grew to two leaf stage the, uniform seedlings were transplanted to opaque plastic containers with 45 cm length, 32 cm width and 15 cm height. Each container contains 60 plants. The plastic containers were shifted to greenhouse conditioned with 16 h light/8 h dark, 18°C day/8.5°C night temperature. The containers were filled with modified Hoagland nutrient solution, with NH_4_^+^ or NO_3_^-^ as the sole N source. Composition of the sole NO_3_^-^ source solution was as follows; the macronutrients (mM): 5.0 N in the forms of Ca(NO_3_)_2_ and KNO_3_, 3.0 K in the forms of KH_2_PO_4_ and KNO_3_, 1.5 Ca as Ca(NO_3_)_2_, 1.0 Mg as MgSO_4_, 1.0 P as KH_2_PO_4_, 0.5 Na as NaCl and micronutrients (mM): 1.0 Fe as Fe-EDTA, 9.10×10^−3^ Mn as MnSO_4_, 0.15×10^−3^ Zn as ZnSO_4_, 0.16×10^−3^ Cu as CuSO_4_, 18.5×10^−3^ B as H_3_BO_3_, 0.52×10^−3^ Mo as MoO_3_. The composition of the sole NH_4_^+^-source solution was: 5.0 N as (NH_4_)_2_SO_4_, 3.0 K as KH_2_PO_4_ and K_2_SO_4_, 1.5 Ca as CaCl_2_ and CaSO_4_, 1.0 Mg as MgSO_4_, 1.0 P as KH_2_PO_4_, 0.5 Na as NaCl and micronutrients were the same with NO_3_^-^-source-solution. The solution pH under each treatment of NH_4_^+^ and NO_3_^-^ was adjusted to 5.5, by titration with 0.1 mM H_2_SO_4_ or 0.1 mM NaOH. To keep the N concentration in the nutrient solutions constant, the nutrient solution was replaced with 3 days interval, and continuously aerated to prevent anoxic conditions.

After 10 days, the wheat seedlings grown in NO_3_^-^ or NH_4_^+^ nutrition were respectively divided into three batches. One batch was treated with 5 mM NO_3_^-^ or 5 mM NH_4_^+^ nutrition as the sole nutrition. Another batch was treated with 5 mM NO_3_^-^ or 5 mM NH_4_^+^ nutrition each combined with 1 μM L-methionine sulfoximine (MSO), which was available to inhibit glutamine synthetase (GS) activity, according to the method proposed by Hirano [29]. The third batch was treated with 5 mM NO_3_^-^ or 5 mM NH_4_^+^ nutrition and each combined with 1 mM glutamate according to the method given by Walch-Liu [30]. The experiment was arranged in a completely randomized block design, and replicated thrice. There were 24 containers for each replication.

### Plant sampling

Shoot and root samples were collected on third day after applying MSO or glutamate treatments, respectively. On the same day, gas exchange measurements were also done. A batch of plants was sampled and splited into two parts; one part plants were used to collect the shoots and roots samples, while other part plants were frozen in liquid nitrogen and stored at -80°C for chemical analysis. Another sample of five plants was taken for dry weight measurements. These plants were divided into shoots and roots and oven dried at 105°C for 20 min and then dried at 75°C to obtain a constant weight.

### Physiological and growth measurements

#### Soluble sugar analysis

Dry powdered shoot and root samples of 0.5 g was extracted in 80°C ethanol (80%) for 30 min. The extracts were centrifuged at 3000 × *g* and supernatant was collected. The extraction procedure was repeated thrice to ensure the complete extraction of soluble sugar from the sample. The collected supernatant was evaporated on a china dish in a hot water bath until dried completely. The residue was re-dissolved in 1–3 mL distilled water and then filtered through 0.4 μm filter film for assay of soluble sugar contents. Content of soluble sugar was measured using the anthrone reagents method. Five mL anthrone sulphuric acid solution (75% v/v) was added to 0.1 mL supernatant and boiled at 90°C for 15 min. Absorbance at 620 nm was read using a spectrophotometer (UV-2401, Shimadzu Corp., Japan).

#### Glutamate extraction and quantification

The frozen shoot and root samples were freeze dried in Virtis Freeze Dryer (−55°C; Gardiner, NY, USA). The dried samples were put into hydrolysis tube and hydrolyzed with 6 mM HCl at 110°C for 24 h. The hydrolysate was dried, dissolved in 0.02 mM HCl and centrifuged at 10,000 rpm for 15 min. The glutamate contents were then obtained by automatic analysis algorithm of the amino acid automatic analyzer. The amino acid analyzer (HITACHI L-8900, Japan) attached HITACHI HPLC Packed Column with Ion-exchanging Resin No. 2622 PF (4.6 mm × 60 mm) and UV detector (VIS1: 570 nm, VIS2: 440 nm) was used for analysis of amino acids. Wako L-8500 buffer solution PF-1, 2, 3, 4 and RG were used in this study. About 20 μL of each sample was injected. Amino acid determination was performed using Ninhydrin reagent set (Wako Chemical Inc, Japan). All samples were run in triplicates.

#### Enzymatic analysis

Glutamine synthetase (GS) and glutamate dehydrogenase (NADH-GDH and NAD-GDH) activities were measured according to the methods of Setien [18] with some modification. Frozen shoot and root samples of 0.5 g with three replications were homogenized in extraction buffer (phosphate buffer, pH 7.2). Extracts were centrifuged at 10,000 × g for 20 min, and supernatant was collected and assayed for the enzymatic activities. The GS activity was measured with reaction buffer (0.5 mol L^-1^ MgSO_4_, 0.3 mol L^-1^ glutamic natrium, 0.03 mol L^-1^ ATP-Na, 0.25 mol L^-1^ imidazole- HCl, and 1 mol L^-1^ hydroxylamine-HCl, pH 7.0) and then incubated at 25°C for 20 min.The reaction was stopped by adding stop solution containing 0.122 M FeCl_3_, 0.5 M TCA and 2 N HCl. Then samples were centrifuged at 10,000 × g for 10 min, and g-glutamyl hydroxymate (γ-GHM) formation was measured spectrophotometrically at 540 nm.

Glutamate dehydrogenase aminating enzyme (NADH-GDH) activity was determined in a reaction buffer containing 115.4 mM Tris–HCl (pH 8.0), 23.1 mM α-ketoglutarate,231 mM NH_4_C1, 30 mM CaCl_2_ and 6 mM coenzyme NADH. While, GDH deaminating enzyme (NAD^+^-GDH) activity was determined in a reaction buffer containing115.4 mM Tris–HCl (pH 9.3), 115.4 mM L-glutamate, 30 mM CaCl_2_ and 30 mM coenzyme NAD. Both kinetic activities were monitored using spectrophotometer at 340 nm at 30°C. One unit of GDH activity was defined by reduction of 1 mM NADH (NADH-GDH) min^-1^ or oxidation of 1 mM NADH (NAD^+^-GDH) min^-1^ at 30°C.

To measure glutamic oxalacetic transaminase (GOT) or glutamic-pyruvic transaminase (GPT) activity, 0.2 g frozen root and shoot samples were homogenized at 4°C in 0.05 M Tris-HCl extraction buffer (pH 7.2, 0.05 M trihydroxymethyl aminomethane was used). The crude extract was centrifuged at 20,000 × g for 20 min and the supernatant was assayed for the enzymatic activities. The GOT activity was measured at 37°C in a reaction buffer containing 200 mM DL-aspartate and 2 mM α-ketoglutarate. The reaction was stopped with 0.5ml 2, 4-dinitrophenylhydrazine, and the absorbance of pyruvic acid was measured at 500 nm. The GPT activity was measured at 37°C in a reaction buffer containing 200 mM DL-alanine, 2 mM α-ketoglutarate, 30 min later adding 0.5 mL 2, 4-dinitrophenylhydrazine to stop the reaction, and the absorbance of pyruvic acid was measured at 500 nm.

#### Phytohormones analysis

Indole-3-acetic acid (IAA) contents were measured using ultra high performance liquid chromatography (UPLC) technique [31] with slightly modification. For sample extraction and purification, the frozen plant material (0.5 g) was put on the pre-cooled mortar, added 5 mL pre-cooled 80% chromatography methanol (v/v) and grinded into pulp in the ice bath to get crude hormone extract. The crude extract was put at 4°C for 12 h then centrifuged at 10,000 × *g* for 10 min and the supernatant was stored in the refrigerator at 4°C. The above extraction process was repeated two times, and all extract was collected, then added PVPP into the extract by 0.2 g/g FW to adsorpt phenolic compounds and pigment, shaking at 4°C for 1 h, then centrifuged at 10,000 × *g* for 10 min. The supernatant was slowly filtrated using C18 column (Sep-Pak C18, Agilent, USA), then poured the outflow into a 50 mL beaker, and dried it in the freeze dryer (Thermo Scientific Heto PowerDry LL3000, USA) at -60°C in dark, then dissolved it adding 5 mL chromatography methanol, at last filter the liquid into 2 mL sample bottles using 0.45 μm organic ultra filtration membrane, to be measured.

Chromatographic column was Eclipse Plus C18 (2.1 × 50 mm, 1.8 Micron, Agilent, USA); The mobile phase: Methanol and ultrapure water (add 0.6% acetic acid); column temperature was 35°C; The sample volume was 2 μL; the flow rate of 0.6 mL/min; detection wavelength was 254 nm.

### Statistical analysis

Analysis of variance (ANOVA) was performed by applying the General Linear Model procedure to calculate the effects of the treatments and cultivars on the investigated morphological and physiological parameters for each sampling and measurement point. Means were compared by using Duncan’s multiple comparison test (P <0.05) to see the level of significance of the variables by using the SPSS statistical package (SPSS Inc., Chicago, IL, USA). Figures were plotted by using Sigma Plot 10.0 software (Systat Software Inc., Chicago, IL, USA).

## Results

### Plant dry weight

NH_4_^+^ treatments significantly reduced plant dry biomass in both cultivars when compared with the 5 mM NO_3_^-^ treatments as shown in Table 1. In AK58, NH_4_^+^-fed plants produced 23.9% and 38.9% lower shoot and root biomass, respectively than NO_3_^-^-fed plants. While for Xumai25, the decrease in the biomass of NH_4_^+^-fed plants with regard to NO_3_^-^-fed plants was only 12.0% in shoots and 18.6% in roots. As a consequence, wheat plants showed a significantly higher shoot: root (S: R) ratio under NH_4_^+^ nutrition. Moreover, the total plant biomass decrease in NH_4_^+^-fed plants for AK58 with respect to NO_3_^-^ nutrition was 28.6%, while, for Xumai25, this decrease was only 14.2%. The plant dry biomass significantly increased in the presence of 1 μM MSO than in its absence under NH_4_^+^ conditions, but MSO had little effect on the growth of plant grown under NO_3_^-^ conditions. On the contrary, the plant dry biomass of two cultivars was markedly lower after adding glutamate, and under NO_3_^-^ condition, the decreases were 17.9% in AK58 and 15.8% in Xumai25, while the dry matter inhibition effect was more obvious under NH_4_^+^ condition accompanied by the application of glutamate. The decreases were 40.2% in AK58, 28.3% in Xumai25, compared with the 5 mM NO_3_^-^ condition. These results indicate that the NH_4_^+^-form nutrition significantly reduced the plant dry biomass in both cultivars, but the effects were less pronounced in Xumai25 than in AK58. The application of MSO could relieved the inhibited growth effects induced by NH_4_^+^-form nutrition, but the application of glutamate made the inhibition effect more serious.

**Table 1:** Dry biomass (mg plant^-1^) of shoot and root and their ratio in wheat plants grown underNO_3_^-^ or NH_4_^+^ conditions with applying MSO or glutamate in AK58 and Xumai25.

	AK58				Xumai25			
Treatment	Shoot dry biomass	Root dry biomass	Plant dry biomass	Shoot/Root	Shoot dry biomass	Root dry biomass	Plant dry biomass	Shoot/Root
**NO**_**3**_^**-**^	232.9±6.1a	104.7±2.1a	337.6±8.2a	2.23±0.01c	256.9±2.4a	124.7±3.5a	381.6±1.1a	2.06±0.08c
**NO**_**3**_^**-**^-**MSO**	228.7±8.0a	103.3±3.5a	332.0±11.5a	2.21±0.01c	254.4±2.6a	120.7±0.7ab	375.1±3.3a	2.11±0.01bc
**NO**_**3**_^**-**^-**Glu**	195.8±2.4b	81.1±2.1c	277.0±0.3b	2.41±0.09bc	225.8±2.4c	95.1±0.7c	321.0±3.1c	2.37±0.01ab
**NH**_**4**_^**+**^	177.1±1.0c	64.0±4.9d	241.1±4.0c	2.78±0.23ab	226.1±1.0c	101.5±1.4c	327.6±0.4c	2.23±0.04bc
**NH**_**4**_^**+**^**-MSO**	196.2±3.7b	94.2±0.7b	290.4±4.4b	2.08±0.02c	242.6±1.7b	113.0±0.7b	355.6±2.4b	2.15±0.01bc
**NH**_**4**_^**+**^**-Glu**	153.6±3.5d	48.3±2.8e	201.9±0.7d	3.19±0.26a	196.1±4.3d	77.4±9.7d	273.5±14d	2.55±0.26a

Data are expressed as mean ± SE (*n* = 10); Different letters in *the same column* indicate significance at *P* < 0.05. NO_3_^-^ refers to nitrate-fed plants; NO_3_^-^-MSO refers to nitrate-fed with the application of MSO; NO_3_^-^-Glu refers to nitrate-fed with the application of glutamate; NH_4_^+^refers to ammonium-fed plants; NH_4_^+^-MSO refers to ammonium-fed with the application of MSO; NH_4_^+^-Glu refers to ammonium-fed with the application of glutamate.

### Root morphology

Under NH_4_^+^ conditions, the total root length, root surface area and root volume decreased distinctly compared with the NO_3_^-^-fed plants in both cultivars (Table 2), but these parameters decreased more significantly in AK58. Compared with the NO_3_^-^nutrition, the total root length, root surface area and root volume were reduced by 29.2%, 32.6% and 33.3%, respectively in AK58, whereas in Xumai25, these were reduced by 17.1%, 20.3% and 17.1%, respectively. However, the total root length, root surface area and root volume were increased by the application of MSO under NH_4_^+^ conditions, but were significantly decreased by the application of glutamate.

**Table 2:** Total root length(cm plant^-1^), surface area (cm^2^ plant^-1^) and root volume (cm^3^ plant^-1^) in wheat plants grown under NO_3_^-^ or NH_4_^+^conditions with applying MSO or glutamate in AK58 and Xumai25.

	AK58			Xumai25		
Treatment	Total root length	Root surface area	Root volume	Total root length	Root surface area	Root volume
**NO**_**3**_^**-**^	466±25a	55.5±2.4a	0.3±0.016a	545±25a	66.7±3.8a	0.35±0.008a
**NO**_**3**_^**-**^-**MSO**	448±16a	53.0±1.7a	0.29±0.009a	517±17ab	65.0±1.9a	0.34±0.012ab
**NO**_**3**_^**-**^-**Glu**	363±19c	43.7±2.3b	0.21±0.009c	394±15d	46.2±3.1b	0.27±0.014c
**NH**_**4**_^**+**^	330±16d	37.4±1.3c	0.2±0.012c	452±17c	53.1±2c	0.29±0.006c
**NH**_**4**_^**+**^**-MSO**	414±25b	51.7±2.5a	0.26±0.007b	499±22b	61.2±4.1a	0.32±0.009b
**NH**_**4**_^**+**^**-Glu**	278±16e	26.9±2.2d	0.16±0.017d	341±32e	39.8±1.5d	0.23±0.014d

Data are expressed as mean ± SE (*n* = 10); Different letters in *the same column* indicate significance at *P* < 0.05. NO_3_^-^ refers to nitrate-fed plants; NO_3_^-^-MSO refers to nitrate-fed with the application of MSO; NO_3_^-^-Glu refers to nitrate-fed with the application of glutamate; NH_4_^+^refers to ammonium-fed plants; NH_4_^+^-MSO refers to ammonium-fed with the application of MSO; NH_4_^+^-Glu refers to ammonium-fed with the application of glutamate.

### Free NH_4_^+^ and glutamate contents

**Fig 1:**
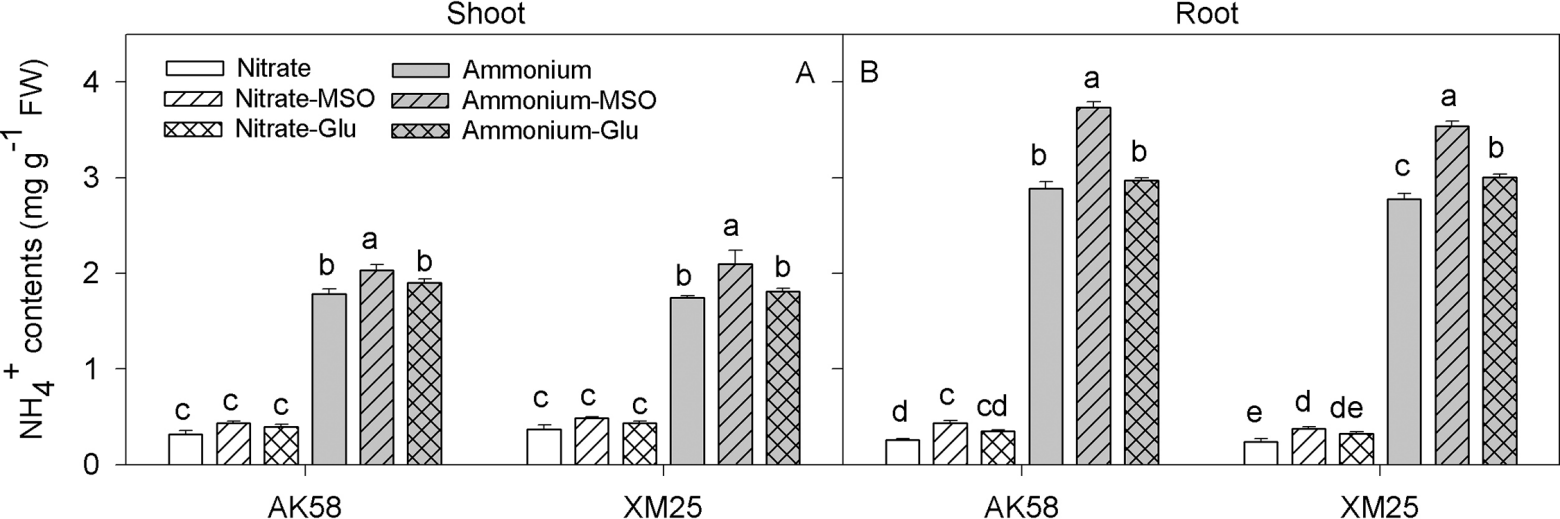
**Free NH_4_^+^ contents in shoots (A) and roots (B) of wheat plants grown under NO**_**3**_^**-**^ **or NH**_**4**_^**+**^ **conditions with applying MSO or glutamate in AK58 (Left) and Xumai25 (Right).** Nitrate refers to nitrate-fed plants; Nitrate-MSO refers to nitrate-fed with the application of MSO; Nitrate-Glu refers to nitrate-fed with the application of glutamate; Ammonium refers to ammonium-fed plants; Ammonium-MSO refers to ammonium-fed with the application of MSO; Ammonium-Glu refers to ammonium-fed with the application of glutamate. Lowercase letters refer to significant difference between treatments (*P*<0.05). Whiskers on the top of the bars indicate standard error (*n* = 6).

The treatment with NH_4_^+^ nutrition induced a sharp increase in free NH_4_^+^ in shoots and roots of both Xumai25 and AK58 (Fig 1). Regardless of the cultivar diversity, the application of MSO to the growth solution resulted in a drastic increase in free NH_4_^+^ content in the seedlings. However, the adding-glutamate treatments seemed had no obvious effects on the accumulation of free NH_4_^+^ except on the root of Xumai25.

**Fig 2:**
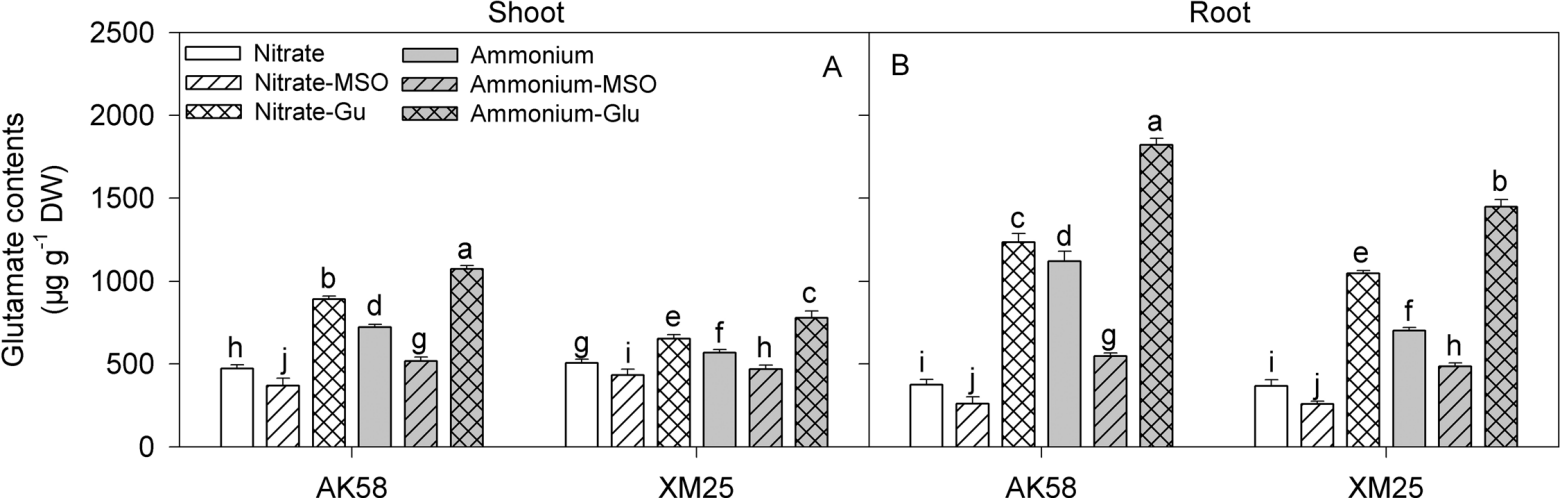
**Glutamate concentrations in shoots (A) and roots (B) of wheat plants grown under NO**_**3**_^**-**^ **or NH**_**4**_^**+**^ **conditions with applying MSO or glutamate in AK58 (Left) and Xumai25 (Right).** Nitrate refers to nitrate-fed plants; Nitrate-MSO refers to nitrate-fed with the application of MSO; Nitrate-Glu refers to nitrate-fed with the application of glutamate; Ammonium refers to ammonium-fed plants; Ammonium-MSO refers to ammonium-fed with the application of MSO; Ammonium-Glu refers to ammonium-fed with the application of glutamate. Lowercase letters refer to significant difference between treatments (*P*<0.05). Whiskers on the top of the bars indicate standard error (*n* = 6).

In both cultivars, glutamate contents were increased significantly under NH_4_^+^ conditions (Fig 2), especially in roots, but the increases were less in Xumai25 than that in AK58. However, after applying the MSO to solution, the glutamate contents were decreased in both shoots and roots, but its contents were significantly increased when glutamate was applied to the solution, especially when it was applied to the NH_4_^+^ solution.

### Glutamine synthetase and glutamate dehydrogenase activities

**Fig 3:**
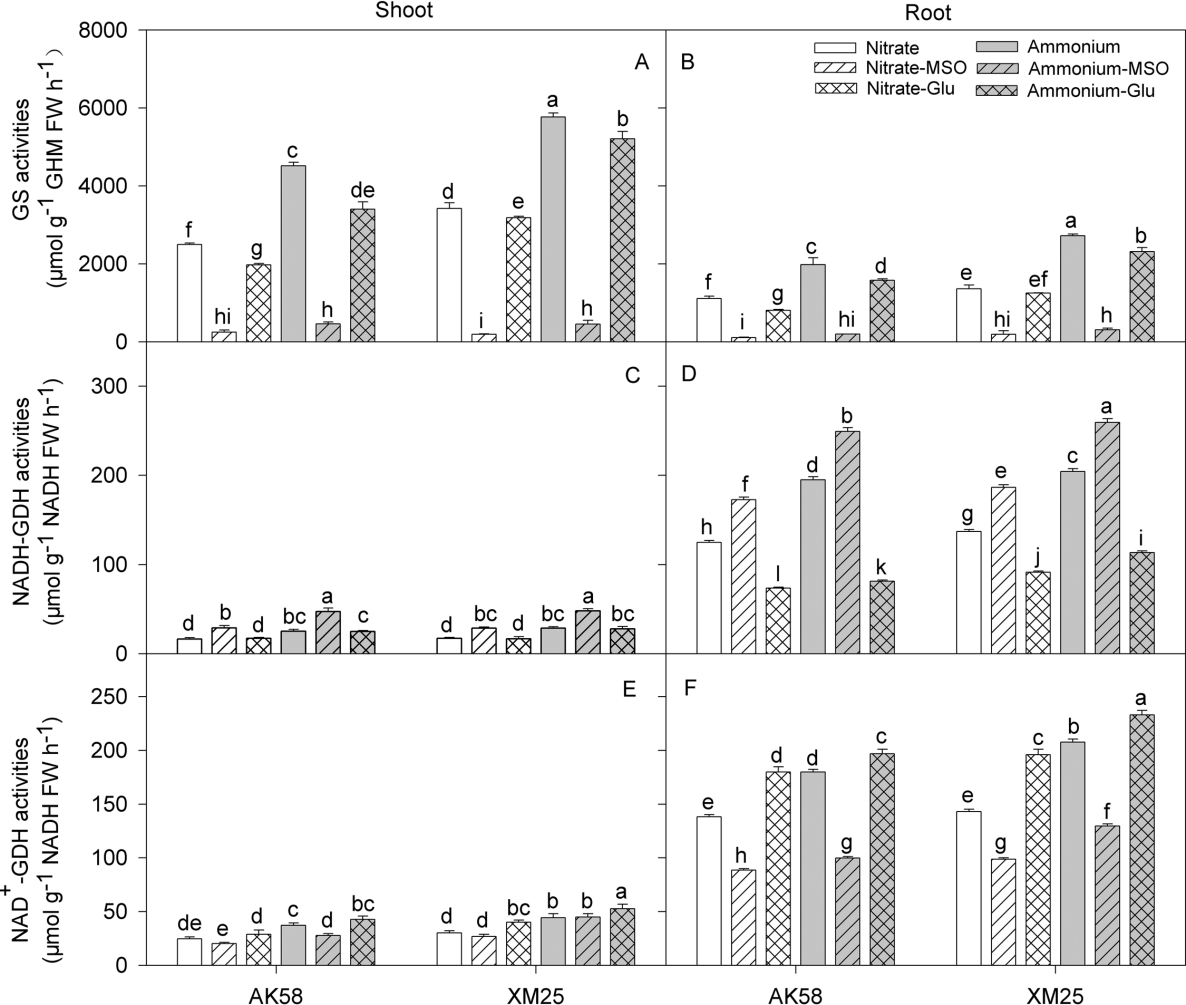
**The activity of glutamine synthetase (GS) (A, B, respectively), glutamate dehydrogenase aminating enzyme (NADH-GDH) (C, D, respectively) and glutamate dehydrogenase deaminating enzyme (NAD**^**+**^**-GDH) (E, F, respectively) in shoots or roots of wheat plants grown under NO**_**3**_^**-**^ **or NH**_**4**_^**+**^ **conditions with applying MSO or glutamate in AK58 (Left) and Xumai25 (Right).** Nitrate refers to nitrate-fed plants; Nitrate-MSO refers to nitrate-fed with the application of MSO; Nitrate-Glu refers to nitrate-fed with the application of glutamate; Ammonium refers to ammonium-fed plants; Ammonium-MSO refers to ammonium-fed with the application of MSO; Ammonium-Glu refers to ammonium-fed with the application of glutamate. Lowercase letters refer to significant difference between treatments (*P*< 0.05). Whiskers on the top of the bars indicate standard error (*n* = 6).

The glutamine synthetase (GS) activity was much higher under NH_4_^+^ condition in both shoots and roots, but it was significantly reduced by the application of MSO in all treatments (Fig 3). The application of glutamate also inhibited GS activity compared to the absence of glutamate in NH_4_^+^ nutrition. Meanwhile, GDH aminating enzyme (NADH-GDH) activity in shoots and roots of NH_4_^+^-fed plants was also increased significantly. With respect to the application of MSO treatments, GDH deaminating enzyme (NAD^+^-GDH) activity were slightly affected in shoots, but its activity was also significantly increased in roots. On the contrary, the application of glutamate inhibited the NADH-GDH activity in both cultivars.

### Glutamic-oxaloacetic transaminase (GOT) and glutamic-pyruvic transaminase (GPT) activities

**Fig 4:**
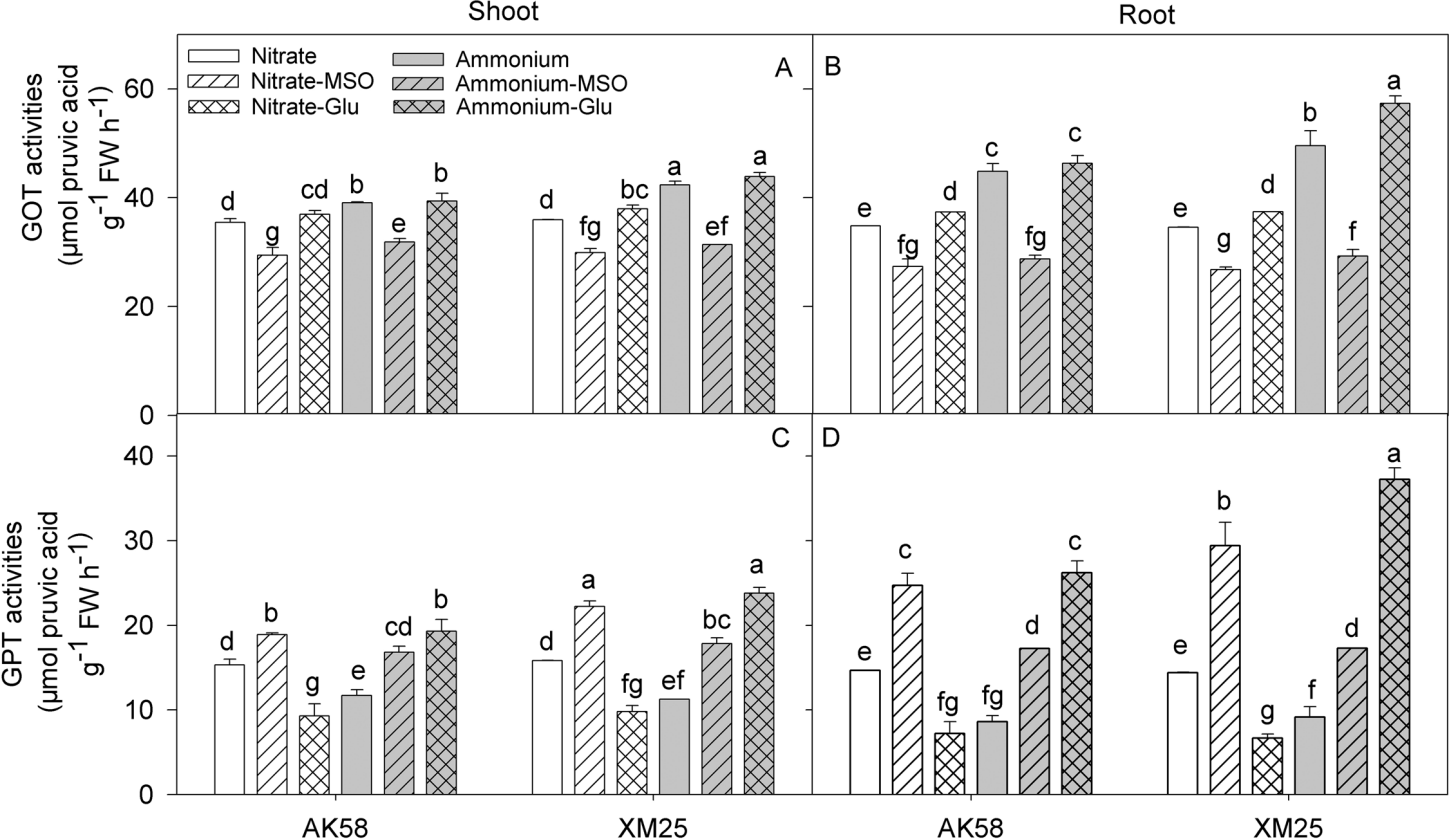
**The activity of glutamic-oxaloacetic transaminase (GOT) (A, B, respectively) and glutamic-pyruvic transaminase (GPT) (C, D, respectively) in shoots or roots of wheat plants grown under NO**_**3**_^**-**^ **or NH**_**4**_^**+**^ **conditions with applying MSO or glutamate in AK58 (Left) and Xumai25 (Right).** Nitrate refers to nitrate-fed plants; Nitrate-MSO refers to nitrate-fed with the application of MSO; Nitrate-Glu refers to nitrate-fed with the application of glutamate; Ammonium refers to ammonium-fed plants; Ammonium-MSO refers to ammonium-fed with the application of MSO; Ammonium-Glu refers to ammonium-fed with the application of glutamate. Lowercase letters refer to significant difference between treatments (*P*< 0.05). Whiskers on the top of the bars indicate standard error (*n* = 6).

The activities of glutamic-oxaloacetic transaminase (GOT) and glutamic-pyruvic transaminase (GPT) were higher under NH_4_^+^ conditions, especially by applying glutamate, but their activities were reduced by the application of MSO. The NH_4_^+^-tolerant cultivar, Xumai25 showed a higher increase in GOT and GPT activity than AK58 under NH_4_^+^ conditions, especially in roots (Fig 4).

### IAA concentrations

**Fig 5:**
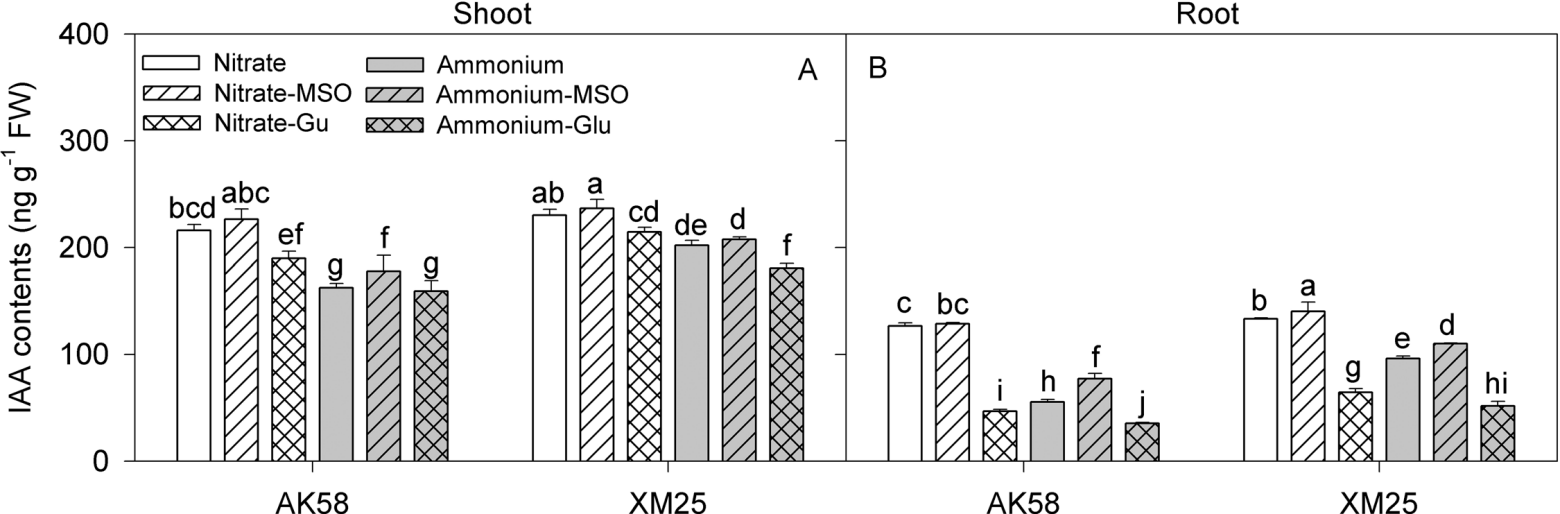
**Indole-3-acetic acid (IAA) concentrations in shoots (A) and roots(B) of wheat plants grown under NO**_**3**_^**-**^ **or NH**_**4**_^**+**^ **conditions with applying MSO or glutamate in AK58 (Left) and Xumai25 (Right).** Nitrate refers to nitrate-fed plants; Nitrate-MSO refers to nitrate-fed with the application of MSO; Nitrate-Glu refers to nitrate-fed with the application of glutamate; Ammonium refers to ammonium-fed plants; Ammonium-MSO refers to ammonium-fed with the application of MSO; Ammonium-Glu refers to ammonium-fed with the application of glutamate. Lowercase letters refer to significant difference between treatments (*P*< 0.05). Whiskers on the top of the bars indicate standard error (*n* = 6).

**Fig 6:**
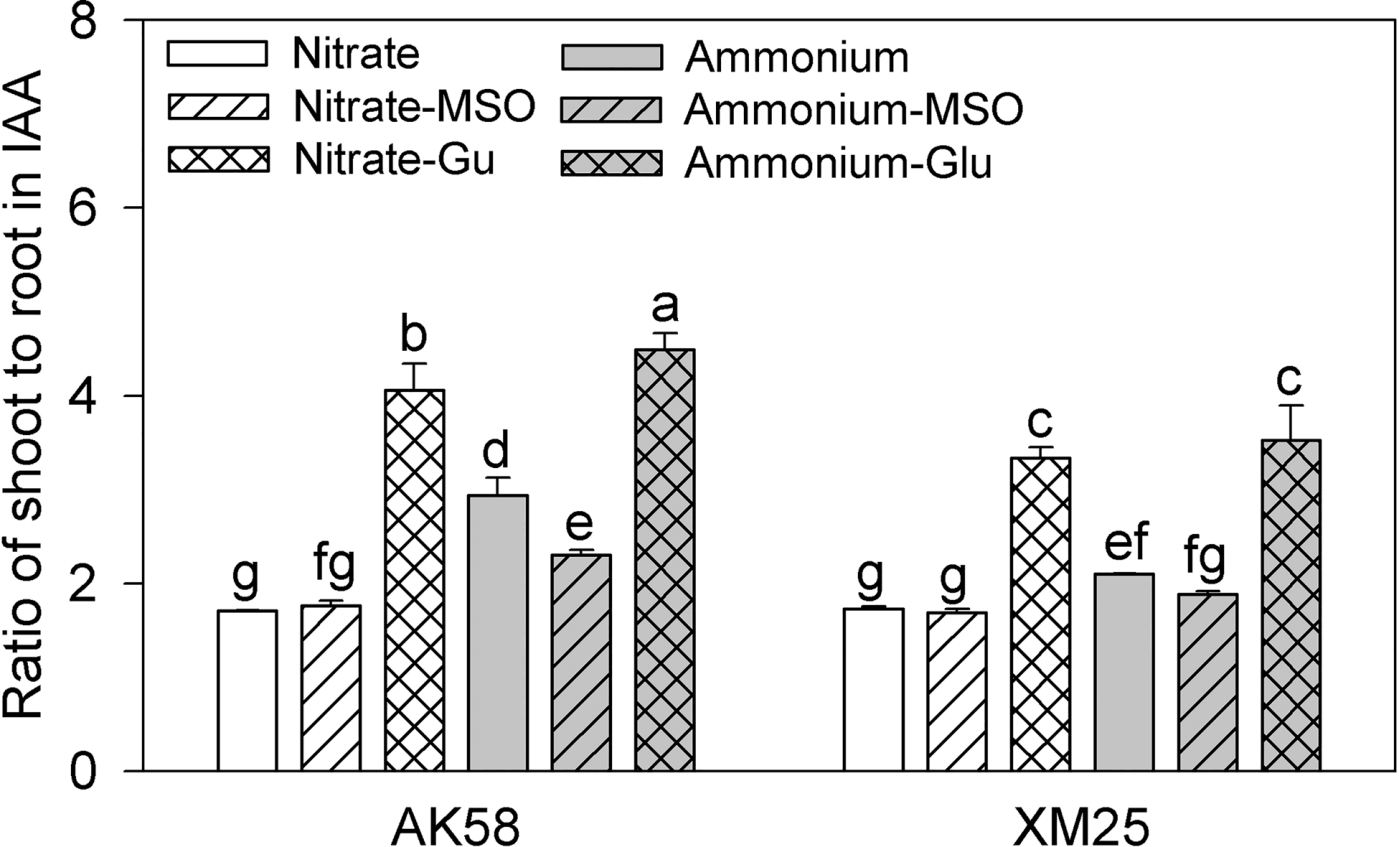
**Ratio of shoot to root in IAA of wheat plants grown under NO**_**3**_^**-**^ **or NH**_**4**_^**+**^ **conditions with applying MSO or glutamate in AK58 (Left) and Xumai25 (Right).** IAA refers to Indole-3-acetic acid. Nitrate refers to nitrate-fed plants; Nitrate-MSO refers to nitrate-fed with the application of MSO; Nitrate-Glu refers to nitrate-fed with the application of glutamate; Ammonium refers to ammonium-fed plants; Ammonium-MSO refers to ammonium-fed with the application of MSO; Ammonium-Glu refers to ammonium-fed with the application of glutamate. Lowercase letters refer to significant difference between treatments (*P*< 0.05). Whiskers on the top of the bars indicate standard error (*n* = 6).

The NH_4_^+^ treatments significantly decreased the IAA concentrations of both cultivars (Fig 5), but the extent of the decrease was much less in Xumai25 compared with AK58. In shoots, the IAA concentrations were decreased by 24.8% in AK58 and 12.2% in Xumai25. In roots, the IAA concentrations were decreased more significantly, which were 56.3% lower in AK58 and 27.8% lower in Xumai25. Hence the ratios of shoot to root in IAA were decreased by 72.1% in AK58, but 21.8% in Xumai25 (Fig 6). In the NH_4_^+^-fed plants, the IAA concentrations got higher by the application of MSO, but reduced more by the application of glutamate in both of shoots and roots,. Meanwhile, the shoot to root ratios of IAA were reduced by the application of MSO but increased by the application of glutamate in the NH_4_^+^-fed plants.

### Soluble sugar contents

Soluble sugar contents were decreased significantly in NH_4_^+^ nutrition compared to NO_3_^-^ nutrition, especially in the roots, but in the NH_4_^+^-tolerant cultivar Xumai25, the reduction was less than that in NH_4_^+^- sensitive ecotype AK58 (Table 3). For Xumai25, under NH_4_^+^conditions, the contents of soluble sugar was about 13.3% lower in shoots and 27.7% lower in roots, in NH_4_^+^-fed plants than in NO_3_^-^-fed plants, but for AK58 this parameters was about 35.7% and 42.2% lower in shoots and roots, respectively. Meanwhile, compared to NO_3_^-^-fed plants, NH_4_^+^-fed plants showed significant higher ratio of shoot to root in soluble sugar, especially inAK58 (Table 3), indicating that the transport of soluble sugar might be interrupted under NH_4_^+^ conditions. By adding MSO to plants in both cultivars the soluble sugar contents increased significantly, but decreased after the application of glutamate. The ratios of shoot to root in soluble sugar were reduced by applying MSO, but increased by the application of glutamate.

**Table 3:** Soluble sugar contents (mg g^-1^ DW) in shoot, root and its ratio of shoot to root in wheat plants grown under NO_3_^-^ or NH_4_^+^ conditions with applying MSO or glutamate in AK58 and Xumai25.

	AK58			Xumai25		
Treatment	Shoot	Root	Shoot/Root	Shoot	Root	Shoot/Root
**NO**_**3**_^**-**^	197±11.5b	66±3.6c	3.0±0.01c	218±8.3b	84±1.0d	2.6±0.07c
**NO**_**3**_^**-**^-**MSO**	205±12.3b	100±5.4a	2.0±0.01d	220±7.2b	120±2.8a	1.8±0.02d
**NO**_**3**_^**-**^-**Glu**	266±4.3a	78±6.2b	3.4±0.33c	289±6.6a	95±1.7c	3.1±0.02b
**NH**_**4**_^**+**^	117±17.5d	29±2.4d	4.0±0.27b	189±3.2c	65±1.7e	2.9±0.03b
**NH**_**4**_^**+**^**-MSO**	120±6.8d	63±4.7c	1.9±0.04d	201±7.0c	104±7.7b	1.9±0.21d
**NH**_**4**_^**+**^**-Glu**	141±2.0c	31±1.8d	4.6±0.20a	157±3.2d	44±2.4f	3.6±0.13a

Data are expressed as mean ± SE (*n* = 10); Different letters in *the same column* indicate significance at *P* < 0.05. Nitrate refers to nitrate-fed plants; Nitrate-MSO refers to nitrate-fed with the application of MSO; Nitrate-Glu refers to nitrate-fed with the application of glutamate; Ammonium refers to ammonium-fed plants; Ammonium-MSO refers to ammonium-fed with the application of MSO; Ammonium-Glu refers to ammonium-fed with the application of glutamate. Lowercase letters refer to significant difference between treatments (*P*< 0.05). Whiskers on the top of the bars indicate standard error (*n* = 6).

## Discussion

It is evident from the results of the study that two wheat cultivars displayed a range of response to NH_4_^+^ stress for regulating their survival, growth and dry biomass production. Our results of variable responses of the cultivars toward NH_4_^+^ stress are in accordance with the studies which have reported the plant response to NH_4_^+^ stress varied significantly among species or even among cultivars within the same species [10,32]. In many previous studies, the most obvious toxicity damages caused by NH_4_^+^ nutrition have been reflected by the reduced plant dry biomass and inhibited root growth [3,33]. For example, Baohai Li [32] found that the shortened and reduced root gravitropism occurred under high NH_4_^+^ conditions in *Arabidopsis* ecotypes, but the ecotype tolerant to NH_4_^+^-toxicity suffered less from NH_4_^+^ toxicity than the sensitive one. Wheat has been described as a species highly sensitive to NH_4_^+^ stress [18]. In the present experiment, the wheat growth was inhibited in both cultivars (Xumai25 and AK58) under NH_4_^+^ conditions, which was indicated by the reduced dry biomass, total root length, surface area and root volume, but the inhibition effect was less in Xumai25 than that in AK58, which indicated that Xumai25 was more tolerant to NH_4_^+^ stress than AK58.

In some previous studies, NH_4_^+^ stress sensitive cultivars were found more NH_4_^+^ accumulation in their tissues than the tolerant cultivars [34]. So, it was considered that NH_4_^+^ toxicity was generally caused by the large amounts of free NH_4_^+^ in plant tissues. NH_4_^+^ is taken up by plant root cells and distributed to the cytosol and some intracellular compartments such as chloroplasts, mitochondria or vacuoles [35]. In some compartments such as vacuoles, the NH_4_^+^ is not metabolized, while cytosol and plastids are the substrates for the NH_4_^+^ metabolization [35]. It was found that when NH_4_^+^ was applied as a sole N source, much of the free NH_4_^+^could be stored in the vacuoles of the underground part cells (roots) to prevent the free NH_4_^+^ transport to the aerial parts, which were considered as more sensitive to NH_4_^+^ stress [10,26]. Under these contexts, the concentrations of free NH_4_^+^ in plant tissues cannot be taken as the only trait conferring NH_4_^+^ tolerance or sensitivity of a plant. In the present study, we made an assumption that NH_4_^+^ tolerance might be associated with the assimilatory metabolism under NH_4_^+^ stress. So we examined the effects of NH_4_^+^ assimilation on wheat growth through the application of MSO (an inhibitor of GS) or glutamate (a primary assimilatory product). Interestingly, for the NH_4_^+^-fed plants, free NH_4_^+^ contents were increased (Fig 1) but the NH_4_^+^-induced inhibitory effect was partially alleviated by the application of MSO, but strengthened by the application of glutamate. This result indicated that apart from the large amounts of free NH_4_^+^ in plant tissues, as a primary N assimilation product, glutamate might also act as a factor involved in the inhibition of root growth under NH_4_^+^stress.

In our study, it was observed that GS activity was higher in NH_4_^+^-fed wheat plants. Similarly, Setién [36] showed that the activity of GS in wheat leaves and roots was significantly higher under NH_4_^+^ condition meanwhile, involving an additional pathway to the GS/GOGAT cycle for the assimilation of NH_4_^+^ into glutamate [24], the aminating-GDH (NADH-GDH) activity was also increased [32]. In some reports, the increased N assimilation enzymes have been considered as a detoxification pathway under high NH_4_^+^ conditions [37], however, it also resulted in a rapid assimilation rate of primary N assimilation products under NH_4_^+^ condition. Actually, higher levels of nitrogenous compounds, such as amino acids, proteins or polyamines were detected when NH_4_^+^ was the sole N source [10,32,36]. In the present experiment, the glutamate contents did get significantly increased by the NH_4_^+^ nutrition, especially in roots, and the effects were more pronounced in AK58 than in Xumai25, indicating that there were some genotypic differences of the cultivars in glutamate metabolism, which might lead to the different levels of glutamate accumulation under NH_4_^+^ stress. In the analysis of glutamic oxalacetic transaminase (GOT; EC 2.6.1.1) and glutamic-pyruvic transaminase (GPT) (EC 2.6.1.2), although the activities of the two transaminases were increased in both cultivars under NH_4_^+^ conditions, but the tolerant-cultivar Xumai25 showed more significant increase than the sensitive-cultivar. So, the higher transamination capacity might be the reason that accounted for its relative lower concentrations of the endogenous glutamate.

Some negative effects of high glutamate contents on plant growth have also been reported [27,30]. Glutamate appeared to be sensed at the root tip, and the mitotic activity in the root tip has been reported as an early target of glutamate inhibition in *Arabidopsis* [30]. Some reports have shown the glutamate inhibited meristematic activity by altering the distribution of IAA in the root tip [30]. Indole-3-acetic acid (IAA) plays important roles in the development of roots and can affects cell division and elongation in root meristem [38,39]. Under NH_4_^+^ stress, cell expansion can even account for about 70% of the inhibition of root elongation induced by NH_4_^+^ [3]. Under NH_4_^+^ conditions, in some auxin-resistant mutants, it was found that the root length is less affected by high NH_4_^+^ concentrations compared with wild type [3], indicating that IAA was tightly linked to NH_4_^+^-induced inhibition of root growth. Consistently, in the present study, IAA contents were decreased significantly in roots, and AK58 showed a greater decrease than Xumai25, which might be responsible for the decreased root growth. It was found that the high endogenous glutamate contents might act as the inhibitory factor in IAA transport from shoots to roots [30]. The reduction of long-distance IAA transport was mediated by AUX1, an IAA influx facilitator participating in IAA transport [3,30]. Under NH_4_^+^conditions, accumulation of endogenous glutamate might repress AUX1 function, resulting in reduction of IAA transport from shoots to roots [3]. We observed that IAA contents were decreased more significantly in roots than in shoots, and higher ratio of shoot to root for IAA was found under NH_4_^+^conditions (Fig 5). Moreover, the ratio was decreased by the application of MSO and increased by the application of glutamate in the NH_4_^+^-fed plants. It proved that long-distance transport of IAA might be interrupted by glutamate from shoots to roots under NH_4_^+^ conditions.

Several previous studies have reported that the root vascular tissues differentiation and regeneration were induced and controlled by polar IAA movement from the young leaves to the root tips [38,40]. Moreover, higher IAA concentration in roots promoting a more active sucrose metabolism was observed in a wheat cultivar Luohan 7 [41]. So, some report found that the carbohydrates transport could be regulated by IAA distribution in plants and increased IAA in roots could promote sucrose transport from leaves to roots. Since N assimilation occurs vastly in roots when NH_4_^+^ is the sole N source, and NH_4_^+^-fed plants roots may act as a stronger sink for carbohydrates [42,43]. An improvement in carbohydrate transport functions from shoots to roots is necessary to ensure a sufficient supply of carbohydrates for the roots growth. In the present study, lower soluble sugar contents were detected in the NH_4_^+^-fed plants, which was in accordance with the previous findings [12,19]. The NH_4_^+^- sensitive cultivar AK58 showed higher ratios of shoot to root in soluble sugar than NH_4_^+^-tolerant cultivar Xumai25, indicating that carbohydrate transport functions of AK58 was weaker than Xumai25. In line with the trend of IAA, the application of MSO could increase the soluble sugar contents in roots and decrease the ratios of shoot to root, while the application of glutamate had the opposite effect. These results suggested that the interrupted IAA transport by glutamate could further interrupt carbohydrate transport, which is essential for root growth.

In conclusions, under NH_4_^+^ conditions, due to the increased N assimilation rate, a large amount of glutamate accumulation in wheat tissues, especially in roots was observed, which resulted in an interruption of IAA transport from shoots to roots, and a sequent reduction of carbohydrate distributed to roots, which inhibited root growth. However, the cultivar Xumai25 showed more capability to keep the lower level of glutamate in roots due to its higher transamination capacity as well as possessing a greater ability to maintain root growth than AK58. Analyses using molecular biology and mutants to explain the interactions between auxin signaling and glutamate signaling are needed for further understandings in the evaluation of genotypic characteristics towards high NH_4_^+^ stress conditions.

## References

[1] Chen G, Guo S, Kronzucker HJ, Shi W (2013) Nitrogen use efficiency (NUE) in rice links to NH4+ toxicity and futile NH4+ cycling in roots. Plant and Soil: 1–13.

[2] Britto DT, Kronzucker HJ (2013) Ecological significance and complexity of N-source preference in plants. Annals of Botany 112: 957–963. 10.1093/aob/mct157 23884397 PMC3783226

[3] Li B, Li G, Kronzucker HJ, Baluška F, Shi W (2014) Ammonium stress in Arabidopsis: signaling, genetic loci, and physiological targets. Trends Plant Sci 19: 107–114. 10.1016/j.tplants.2013.09.004 24126103

[4] Xu G, Fan X, Miller AJ (2012) Plant nitrogen assimilation and use efficiency. Annu Rev Plant Biol 63: 153–182. 10.1146/annurev-arplant-042811-105532 22224450

[5] Shi Z, Li D, Jing Q, Cai J, Jiang D, Cao W, et al (2012) Effects of nitrogen applications on soil nitrogen balance and nitrogen utilization of winter wheat in a rice-wheat rotation. Field Crops Research 127: 241–247.

[6] Ariz I, Esteban R, Ignacio Garcia-Plazaola J, Maria Becerril J, Maria Aparicio-Tejo P, Fernando Moran J (2010) High irradiance induces photoprotective mechanisms and a positive effect on NH4+ stress in Pisum sativum L. Journal of Plant Physiology 167: 1038–1045. 10.1016/j.jplph.2010.02.014 20434233

[7] Guo J, Liu X, Zhang Y, Shen J, Han W, Zhang W, et al (2010) Significant acidification in major Chinese croplands. Science 327: 1008–1010. 10.1126/science.1182570 20150447

[8] Van Katwijk M, Vergeer L, Schmitz G, Roelofs J (1997) Ammonium toxicity in *eelgrass Zostera* marina. Marine Ecology Progress Series 157: 159–173.

[9] Peckol P, Rivers J (1995) Physiological responses of the opportunistic macroalgae *Cladophora vagabunda* (L.) van den Hoek and *Gracilaria tikvahiae* (McLachlan) to environmental disturbances associated with eutrophication. Journal of Experimental Marine Biology and Ecology 190: 1–16.

[10] Britto DT, Kronzucker HJ (2002) NH4+ toxicity in higher plants: a critical review. Journal of Plant Physiology 159: 567–584.

[11] Aloni R, Aloni E, Langhans M, Ullrich C (2006) Role of cytokinin and auxin in shaping root architecture: regulating vascular differentiation, lateral root initiation, root apical dominance and root gravitropism. Annals of Botany 97: 883–893. 16473866 10.1093/aob/mcl027 PMC2803412

[12] Ariz I, Asensio AC, Zamarreno AM, Garcia-Mina JM, Aparicio-Tejo PM, Moran JF (2013) Changes in the C/N balance caused by increasing external ammonium concentrations are driven by carbon and energy availabilities during ammonium nutrition in pea plants: the key roles of asparagine synthetase and anaplerotic enzymes. Physiologia Plantarum 148: 522–537. 10.1111/j.1399-3054.2012.01712.x 23061733

[13] Taylor MW, Taylor RB, Rees TAV (1999) Allometric evidence for the dominant role of surface cells in ammonium metabolism and photosynthesis in northeastern New Zealand seaweeds. Marine Ecology Progress Series 184: 73–81.

[14] Tong D, Xu R (2012) Effects of urea and (NH_4_)_2_SO_4_ on nitrification and acidification of Ultisols from Southern China. Journal of Environmental Sciences-China 24: 682–689. 22894103 10.1016/s1001-0742(11)60832-2

[15] Van Den Berg LJ, Dorland E, Vergeer P, Hart MA, Bobbink R, Roelofs JG (2005) Decline of acid‐sensitive plant species in heathland can be attributed to ammonium toxicity in combination with low pH. New Phytologist 166: 551–564. 15819917 10.1111/j.1469-8137.2005.01338.x

[16] Yousra M, Akhtar J, Saqib ZA, Saqib M, Haq MA (2013) Effect of potassium application on ammonium nutrition in maize (*Zea mays* L.) under salt stress. Pakistan Journal of Agricultural Sciences 50: 43–48.

[17] Sebastian A, Prasad MNV (2014) Photosynthesis mediated decrease in cadmium translocation protect shoot growth of *Oryza sativa* seedlings up on ammonium phosphate—sulfur fertilization. Environmental Science and Pollution Research 21: 986–997. 10.1007/s11356-013-1948-7 23852466

[18] Setien I, Fuertes-Mendizabal T, Gonzalez A, Ma Aparicio-Tejo P, Gonzalez-Murua C, Begona Gonzalez-Moro M, et al (2013) High irradiance improves ammonium tolerance in wheat plants by increasing N assimilation. Journal of Plant Physiology 170: 758–771. 10.1016/j.jplph.2012.12.015 23485260

[19] Ariz I, Artola E, Cabrera Asensio A, Cruchaga S, Maria Aparicio-Tejo P, Fernando Moran J (2011) High irradiance increases NH4+ tolerance in *Pisum sativum*: Higher carbon and energy availability improve ion balance but not N assimilation. Journal of Plant Physiology 168: 1009–1015. 10.1016/j.jplph.2010.11.022 21371777

[20] Zou N, Li B, Dong G, Kronzucker HJ, Shi W (2012) Ammonium-induced loss of root gravitropism is related to auxin distribution and TRH1 function, and is uncoupled from the inhibition of root elongation in Arabidopsis. J Exp Bot 63: 3777–3788. 10.1093/jxb/ers068 22407650

[21] Britto DT, Siddiqi MY, Glass ADM, Kronzucker HJ (2001) Futile transmembrane NH4+ cycling: A cellular hypothesis to explain ammonium toxicity in plants. Proceedings of the National Academy of Sciences of the United States of America 98: 4255–4258. 11274450 10.1073/pnas.061034698 PMC31212

[22] El Omari R, Rueda-López M, Avila C, Crespillo R, Nhiri M, Cánovas FM (2010) Ammonium tolerance and the regulation of two cytosolic glutamine synthetases in the roots of sorghum. Functional Plant Biology 37: 55–63.

[23] Bernard SM, Moller ALB, Dionisio G, Kichey T, Jahn TP, Dubois F, et al (2008) Gene expression, cellular localisation and function of glutamine synthetase isozymes in wheat (*Triticum aestivum* L.). Plant Molecular Biology 67: 89–105. 10.1007/s11103-008-9303-y 18288574

[24] Zhou Y, Liu H, Zhou X, Yan Y, Du C, Li Y, et al (2014) Over-expression of a fungal NADP(H)-dependent glutamate dehydrogenase PcGDH improves nitrogen assimilation and growth quality in rice. Molecular Breeding 34: 335–349.

[25] Miflin BJ, Habash DZ (2002) The role of glutamine synthetase and glutamate dehydrogenase in nitrogen assimilation and possibilities for improvement in the nitrogen utilization of crops. J Exp Bot 53: 979–987. 11912240 10.1093/jexbot/53.370.979

[26] Cruz C, Bio AFM, Dominguez-Valdivia MD, Aparicio-Tejo PM, Lamsfus C, Martins-Loucao MA (2006) How does glutamine synthetase activity determine plant tolerance to ammonium? Planta 223: 1068–1080. 16292661 10.1007/s00425-005-0155-2

[27] Forde BG, Lea PJ (2007) Glutamate in plants: metabolism, regulation, and signalling. J Exp Bot 58: 2339–2358. 17578865 10.1093/jxb/erm121

[28] Zhu Y, Di T, Xu G, Chen X, Zeng H, Yan F, et al (2009) Adaptation of plasma membrane H^+^-ATPase of rice roots to low pH as related to ammonium nutrition. Plant Cell and Environment 32: 1428–1440. 10.1111/j.1365-3040.2009.02009.x 19558410

[29] Hirano T, Satoh Y, Ohki A, Takada R, Arai T, Michiyama H (2008) Inhibition of ammonium assimilation restores elongation of seminal rice roots repressed by high levels of exogenous ammonium. Physiologia Plantarum 134: 183–190. 10.1111/j.1399-3054.2008.01117.x 18419739

[30] Walch-Liu P, Liu L-H, Remans T, Tester M, Forde BG (2006) Evidence that L-glutamate can act as an exogenous signal to modulate root growth and branching in *Arabidopsis thaliana*. Plant and Cell Physiology 47: 1045–1057. 16816406 10.1093/pcp/pcj075

[31] Pan X, Welti R, Wang X (2010) Quantitative analysis of major plant hormones in crude plant extracts by high-performance liquid chromatography–mass spectrometry. Nature protocols 5: 986–992. 10.1038/nprot.2010.37 20448544

[32] Li B, Shi W, Su Y (2011) The differing responses of two Arabidopsis ecotypes to ammonium are modulated by the photoperiod regime. Acta Physiologiae Plantarum 33: 325–334.

[33] Kronzucker HJ, Britto DT, Davenport RJ, Tester M (2001) Ammonium toxicity and the real cost of transport. Trends Plant Sci 6: 335–337. 11495764 10.1016/s1360-1385(01)02022-2

[34] Jampeetong A, Brix H, Kantawanichkul S (2012) Response of Salvinia cucullata to high NH4+ concentrations at laboratory scales. Ecotoxicology and Environmental Safety 79: 69–74. 10.1016/j.ecoenv.2011.12.003 22195762

[35] Howitt SM, Udvardi MK (2000) Structure, function and regulation of ammonium transporters in plants. Biochimica Et Biophysica Acta-Biomembranes 1465: 152–170. 10.1016/s0005-2736(00)00136-x 10748252

[36] Setien I, Vega-Mas I, Celestino N, Erendira Calleja-Cervantes M, Gonzalez-Murua C, Maria Estavillo J, et al (2014) Root phosphoenolpyruvate carboxylase and NAD-malic enzymes activity increase the ammonium-assimilating capacity in tomato. Journal of Plant Physiology 171: 49–63. 10.1016/j.jplph.2013.10.021 24484958

[37] Lea PJ, Miflin BJ (2003) Glutamate synthase and the synthesis of glutamate in plants. Plant Physiology and Biochemistry 41: 555–564.

[38] Nishitani K, Masuda Y (1981) Auxin‐induced changes in the cell wall structure: Changes in the sugar compositions, intrinsic viscosity and molecular weight distributions of matrix polysaccharides of the epicotyl cell wall of Vigna angularis. Physiologia Plantarum 52: 482–494.

[39] Bhalerao RP, Eklöf J, Ljung K, Marchant A, Bennett M, Sandberg G (2002) Shoot‐derived auxin is essential for early lateral root emergence in Arabidopsis seedlings. The Plant Journal 29: 325–332. 11844109 10.1046/j.0960-7412.2001.01217.x

[40] Berleth T, Mattsson J, Hardtke CS (2000) Vascular continuity and auxin signals. Trends Plant Sci 5: 387–393. 10973094 10.1016/s1360-1385(00)01725-8

[41] Han H, Tian Z, Fan Y, Cui Y, Cai J, Jiang D, et al (2015) Water-deficit treatment followed by re-watering stimulates seminal root growth associated with hormone balance and photosynthesis in wheat (*Triticum aestivum* L.) seedlings. Plant Growth Regulation 77: 201–210.

[42] Schortemeyer M, Stamp P, Feil B (1997) Ammonium tolerance and carbohydrate status in maize cultivars. Annals of Botany 79: 25–30.

[43] Gerendas J, Zhu ZJ, Bendixen R, Ratcliffe RG, Sattelmacher B (1997) Physiological and biochemical processes related to ammonium toxicity in higher plants. Zeitschrift Fur Pflanzenernahrung Und Bodenkunde 160: 239–251.

